# Stacked kinship CNN vs. GBLUP for genomic predictions of additive and complex continuous phenotypes

**DOI:** 10.1038/s41598-022-24405-0

**Published:** 2022-11-18

**Authors:** Nelson Nazzicari, Filippo Biscarini

**Affiliations:** 1 CREA Council for Agricultural Research and Analysis of Agricultural Economics, Research Centre for Animal Production and Aquaculture, Viale Piacenza 29, 26900 Lodi, Italy; 2 CNR: National Research Council, Institute of Agricultural Biology and Biotechnology, Via Bassini 15, Milan, 20133 Italy

**Keywords:** Genetics, Statistics, Machine learning, Statistical methods

## Abstract

Deep learning is impacting many fields of data science with often spectacular results. However, its application to whole-genome predictions in plant and animal science or in human biology has been rather limited, with mostly underwhelming results. While most works focus on exploring alternative network architectures, in this study we propose an innovative representation of marker genotype data and tested it against the GBLUP (Genomic BLUP) benchmark with linear and nonlinear phenotypes. From publicly available cattle SNP genotype data, different types of genomic kinship matrices are stacked together in a 3D pile from where 2D grayscale slices are extracted and fed to a deep convolutional neural network (DNN). We simulated nine phenotype scenarios with combinations of additivity, dominance and epistasis, and compared the DNN to GBLUP-A (computed using only the additive kinship matrix) and GBLUP-optim (additive, dominance, and epistasis kinship matrices, as needed). Results varied depending on the accuracy metric employed, with DNN performing better in terms of root mean squared error (1–12% lower than GBLUP-A; 1–9% lower than GBLUP-optim) but worse in terms of Pearson’s correlation (0.505 for DNN compared to 0.672 and 0.669 of GBLUP-A and GBLUP-optim for fully additive case; 0.274 for DNN, 0.279 for GBLUP-A, and 0.477 for GBLUP-optim for fully dominant case). The proposed approach offers a basis to explore further the application of DNN to tabular data in whole-genome predictions.

## Introduction

Thanks to innovations in hardware (e.g. GPU/TPU), computing infrastructures (e.g. cloud computing), algorithms (e.g. improved optimizers like RMSProp, Adam), and to the increasing availability of large datasets, deep learning models have emerged as highly successful predictive models in a number of disparate fields, e.g. computer vision, machine translation, sound recognition^1–3^. A neural network becomes *deep* when containing more than one hidden layer. As such, deep learning belongs to the family of machine learning methods, but with some very special characteristics: it is highly suited for non-linear predictive problems, it typically requires large datasets to deploy its potential, and it has a very large set of hyperparameters to explore and fine-tune.

Deep learning has found ample applications to the processing of image and sound data in life-science fields like precision medicine^4^, precision livestock farming^5^ and smart farming^6^. The analysis of non-tabular data is a task at which deep learning excels. However, interest has been given also to its application to model phenotypes, disease risk or breeding values as a function of genomic variants, as in whole-genome predictions^7,8^. This is relevant, for instance, to increase the efficiency and profitability of selection programs in plant and animal breeding, or to accurately predict the genetic susceptibility to specific diseases of individual patients. Current benchmarks in whole-genome predictions are based on variations and different parameterizations of linear models, including genomic best linear unbiased prediction (GBLUP) and the family of Bayesian alphabet models. These approaches have generally proven to be effective for many complex phenotypes under multiple scenarios^9^.

The application of deep learning to whole-genome predictions is relatively recent and has addressed both regression and classification problems, typically feeding a matrix of single nucleotide polymorphism (SNP) genotypes to dense, one-dimensional convolutional neural networks (CNN) or recurrent neural networks. Results so far have been somewhat underwhelming, with performances in general similar to or worse than GBLUP and related models^10^. For regression problems in particular, previous studies failed to find deep learning implementations that outperformed benchmark linear models^11–14^, except for few specific problems (e.g. in polyploid species^15^; for DNA methylation^16^). A telling example comes from a study by Zingaretti et al.^11^ that trained a convolutional network to predict complex phenotype in polyploid species to find results comparable to standard genomic regressions. A very comprehensive, albeit somewhat dated, review^12^ shows that many neural network experiments end up at best with negligible improvements on penalized regression models, and most often not even that. Based on the current literature, CNN architectures seem in general more promising than dense architectures^12^: however, the key to take full advantage of DNN models in whole-genome predictions has yet to be found.

Most published works try to leverage innovative network architectures, often tailored and optimized for a specific problem. In the present work we instead shift the focus on data representation. We propose an approach to reduce input dimensionality that uses kinship matrices to provide a compact transformation of SNP genotype data. Several kinship matrices were stacked together and fed to a 2D-convolutional neural network for the prediction of continuous phenotypes. Additionally, despite its excellent performance on additive traits, GBLUP underperforms with nonliner phenotypes, while DNNs are natural candidates to work with non-linear problems. Therefore, in this study phenotypes were simulated along a complexity gradient: from purely additive phenotypes, to purely dominant phenotypes, including phenotypes generated by a mixture of additive, dominant and epistatic effects. The performance of the developed DNN models was compared to two GBLUP models, namely additive-only GBLUP (GBLUP-A) and GBLUP built using, for each phenotype, the optimal combination of kinship matrices (GBLUP-optim). The aim of the study was to test a novel data representation in DNN models for whole-genome predictions, and to compare it with the GBLUP-benchmark against linear (purely additive) and nonlinear (dominance and epistasis) phenotypes.

## Material and methods

### SNP genotypes

SNP genotype data were publicly available^17^ and consisted of Illumina BovineSNP50 Beadchip genotypes for 1,285 samples from three cattle breeds (1033 Holstein Friesian, 105 Groningen White Headed, 147 Meuse Rhine Yssel). Quality control from the original publication consisted in removing SNPs with call-rate $< 95\%$, with unknown map position or placed on the sex chromosomes. SNPs with minor allele frequency (MAF) $\le 0.5\%$, SNPs for which only two of the three possible genotypes were observed and SNPs in complete linkage disequilibrium (LD, measured as $r^2 = 1$) with an adjacent SNP were also removed. Additional details on data edting can be found in Wientjes et al.^18^. The data made publicly available by the authors consist of the 26,503 SNPs that remained after quality control and editing. To avoid potential issues with population stratification, in present study we used only the 1033 pure-bred Holstein Friesian genotype records. We did not apply any additional SNP data editing.

### Simulated phenotypes

**Figure 1:**
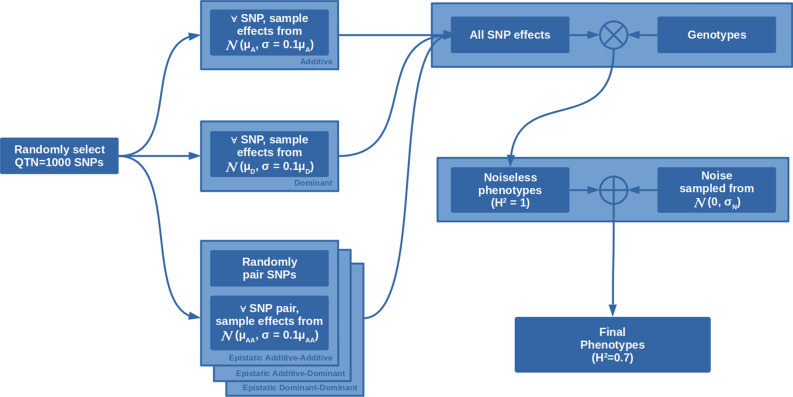
Procedure for simulated phenotypes generation. Effect mean values ($\mu _A, \mu _D, \mu _{AA}, \mu _{AD}, \mu _{DD}$) depend on the simulated scenario. ${\mathcal {N}}(\mu , \sigma )$ is the normal distribution as a function of its mean and standard deviation. $H^2$ is the broad-sense heritability. Noise standard deviation $\sigma _N$ is added after SNP effects are computed so that to guarantee the desired value of $H^2=0.7$.

We simulated phenotypes as the sum of additive, dominant and epistatic effects, plus a noise component to simulate less-than-complete heritability. The effects are linked to a number of markers, selected at random, which in our simulation represent the quantitative trait nucleotides (QTNs).

We started by defining a set of parameters common to all simulations, namely the number of SNPs having an effect ($QTN=1000$), the broad-sense heritability ($H^2=0.7$), the final phenotype mean (equal to zero) and the coefficient of variation for the effect of each QTN ($cv=0.1$). The first two parameters in particular (QTN and $H^2$) were selected to simulate a representative instance of polygenic (complex) traits similar to well known cases in human, animal or plant biology—such as height, biomass or yield—where hundreds of loci contribute to the final phenotypic value^19–23^. Chromosome subdivision was not considered since in whole-genome predictions the matrix of all genomic variants is used irrespective of position^24^.

For each scenario we then assigned a different mean effect to the additive, dominant and epistatic components. We adopted the definition of epistasis as the interaction between two loci. Moreover, each epistatic QTN can behave as an additive or dominant locus. As such there are three possible types of epistasis to be considered: additive–additive (AA), additive–dominant (AD) and dominant–dominant (DD). The fourth possible combination (dominant additive, DA) can be ignored without losing generality since it’s equivalent to AD. Changing the magnitude of these effects allowed us to simulate several phenotype architectures, e.g. the case of a fully additive phenotype, or a desired combination of effects. The considered scenarios are reported in Table 1. For each scenario the final phenotypes were simulated following the procedure illustrated in Fig. 1 and detailed as follows:

1. Randomly select QTN ($=1000$) markers
2. Assign to each selected marker an additive effect, sampling it from a normal distribution with mean $\mu _A$ as reported in Table 1 and standard deviation equal to the same mean multiplied by the coefficient of variability ($cv=0.1$) common to all scenarios. For each marker there is a further 50% chance to have the sign of the effect swapped to negative, to remove biases toward reference/alternative alleles.
3. Same as above, but with dominant effects with mean $\mu _D$
4. Randomly couple the selected markers so that QTN/2 ($=500$) pairs are defined. Assign to each pair effects with means $\mu _{AA}$, $\mu _{AD}$, and $\mu _{DD}$ as described above
5. Multiplying marker effects and genotypes we obtain noiseless phenotypes, which would have perfect heritability.
6. Gaussian noise is added to obtain the target broad sense heritability. The noise variance $\sigma _N^2$ is computed from the genetic variance $\sigma _G^2$ as $\sigma _N^2 = \sigma _G^2 (1-H^2)/H^2$, with $\sigma _G^2$ being computed using Cockerham’s model^25,26^
7. Phenotypes are normalized to zero mean and unitary variance

Note that given that effects are sampled from a probability distribution with fixed coefficient of variation (*cv*), when the assigned mean is equal to zero by definition also the standard deviation goes to zero. In other terms, when the mean of an effect is zero that effect is turned off. With the above procedure, we generated four sets of simulated data (Simulation 0, 1, 2 and 3), each comprising the nine combinations of additive, dominant and epistatic effects (Scenario S1, S2, ..., S9). This means that each of the 9 phenotypes was simulated 4 times, each time being a different sampling instance from the same distribution. Simulation 0, Scenario S1 was used to build the deep learning model; simulations 1, 2 and 3 (with phenotype scenarios S1–S9) were used to test and compare DNN and GBLUP models.

**Table 1:** The simulated phenotype scenarios.

Scenario	Additivity	Dominance	Epistasis			Description
	$\mu _A$	$\mu _D$	$\mu _{AA}$	$\mu _{AD}$	$\mu _{DD}$	
S1	100	0	0	0	0	Fully additive phenotype
S2	75	25	0	0	0	Mixture of additive and dominant effects
S3	50	50	0	0	0	Mixture of additive and dominant effects
S4	25	75	0	0	0	Mixture of additive and dominant effects
S5	0	100	0	0	0	Fully dominant phenotype
S6	33	33	34	0	0	Additive and dominant effects, plus a single epistatic effect
S7	33	33	0	34	0	Additive and dominant effects, plus a single epistatic effect
S8	33	33	0	0	34	Additive and dominant effects, plus a single epistatic effect
S9	33	33	11.3	11.3	11.3	Additive and dominant effects, plus three different epistatic effects

In each scenario a fixed number of SNPs ($QTN=1000$) were randomly selected and assigned additive effects sampled from a normal distribution with mean $\mu _A$, a fixed coefficient of variation ($cv=0.1$) and thus a determined standard deviation ($\sigma = cv\cdot \mu = 0.1\mu$). The operation is repeated for dominant and epistatic effects using the distribution means reported in the corresponding columns. Three types of epistatic effects are considered, when the interacting loci have both additive (AA), both dominant (DD), or additive and dominant (AD) effects.

### Genomic data representation

Several types of kinship matrices were computed from the SNP data to represent different aspects of phenotype architectures. We computed the realized additive kinship ($K_A$) matrix^27^, which summarizes a genetic contribution where each copy of an allele carries an intrinsic value (either positive or negative) to the final phenotype. Thus in case of diploid organisms a homozygous genotype will have twice the effect of a heterozygous genotype for the same locus. We then computed the realized dominance ($K_D$) kinship matrix^28^ following an underlying genetic model where heterozygous and dominant homozygous genotypes have the same effect for a locus. Additive-by-additive ($K_{AA}$), additive-by-dominance ($K_{AD}$) and dominance-by-dominance ($K_{DD}$) realized kinship matrices were obtained by the Hadamard product (element-wise) of the component matrices (e.g. $K_{AA} = K_A \circ K_A$ etc.).

SNP markers were filtered based on their MAF (minor allele frequency). We adopted three filtering strategies, keeping markers with: (1) MAF $> 1\%$; (2) MAF $> 5\%$; (3) MAF in 1–5% (rare alleles only). These three filtering strategies, combined with the five types of genetic relationships ($K_A$, $K_D$, $K_{AA}$, $K_{AD}$, $K_{DD}$), yielded a total of 15 different kinship matrices.

Kinship matrices are square with size $n \times n$, where *n* is the number of samples. The sample order is not relevant when using the matrix for linear regression models. However, this is not the case for convolutional neural networks, which leverage the so called “data locality”. To mimick the properties of neighbouring pixels in actual images we decided to reorder samples so to maximize data locality. The optimal order was obtained applying hierarchical clustering of pairwise dissimilarities^29^ on the 5%-MAF $K_A$ matrix, which was taken as reference. All other matrices were sample-sorted using the same order.

**Figure 2:**
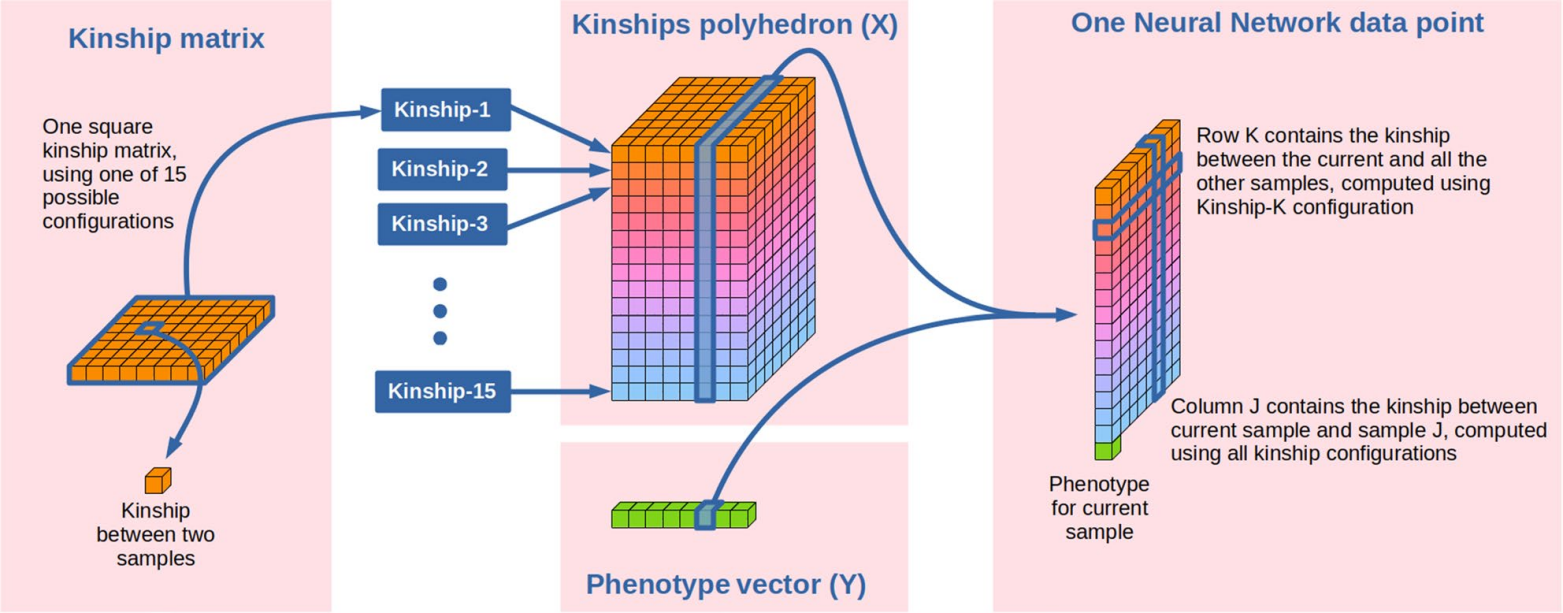
Relationship between continuous phenotypes and SNP genotype data as modeled in this study. SNP genotypes were used to build 15 kinship matrices based on mixture of additive, dominance and epistasis effects. The matrices were then ordered by pairwise similarities and stacked together in a $(n \times n \times 15)$ 3D polyhedron. For each sample one phenotypic value was paired to one $(15 \times n)$ 2D slice of data and fed to the network.

The 15 ordered kinship matrices were then stacked one on top of the other and transposed to give a $(n \times n \times 15)$ 3D polyhedron, as reported in Fig. 2. This data structure was then sliced to obtain *n* slices, one per sample, each dimensioned $n \times 15$. Each of these slice contained information on the relationship between one sample and all the other $n-1$ samples, computed using all fifteen different approaches for kinship computation. Each polyhedron slice was treated as a 2D grayscale images to be fed to the DNN.

### GBLUP models

GBLUP uses genomic relationships to estimate the genetic value of an individual^30^. In its simplest form, the model uses a realized additive kinship matrix as covariance between individuals, as follows:

1 $$\begin{aligned} \left\{ \begin{array}{l} {\textbf{y}} = {\textbf{1}}\mu + \mathbf {Z_a} \mathbf {u_a} + {\textbf{e}} \\ var({\textbf{y}}) = \mathbf {K_a}\sigma ^2_a + {\textbf{I}}\sigma ^2_e \end{array} \right. \end{aligned}$$

where ${\textbf{y}}$ is the vector of phenotypes; $\mu$ is the overall mean; $\mathbf {Z_a}$ is the incidence matrix relating individual samples $u_a$ to *y*; *e* is a vector of residuals; $\mathbf {K_a}$ is the realized additive kinship matrix; ${\textbf{I}}$ is the identity matrix; $\sigma ^2_a$ and $\sigma ^2_e$ are the additive genetic and residual variances; ${\textbf{y}} \sim N(0,\mathbf {K_a}\sigma ^2_a)$ and ${\textbf{e}} \sim N(0,{\textbf{I}}\sigma ^2_e)$. In the rest of the paper this will be referred to as the GBLUP-A model. Model 1 is easily extendable to include different types of kinship matrices, each capturing different links between genotypes and phenotypes. The most comprehensive form is summarized below:

2 $$\begin{aligned} \left\{ \begin{array}{l} {\textbf{y}} = {\textbf{1}}\mu + \left( \sum \limits _{i}^{A, D, AA, AD, DD} \mathbf {Z_i} \mathbf {u_i} \right) + {\textbf{e}} \\ var({\textbf{y}}) = \left( \sum \limits _{i}^{A, D, AA, AD, DD} \mathbf {K_i}\sigma ^2_i \right) + {\textbf{I}}\sigma ^2_e \end{array} \right. \end{aligned}$$

where *A*, *D*, *AA*, *AD*, *DD* refer to the various combinations of additive, dominant and epistatic effects.

It is suboptimal to always include all kinship matrices even when some types of effect are not used in the generation of the simulated phenotypes. We instead decided to include only the matrices corresponding to the effects present in each scenario. E.g, with reference to Table 1, in scenario S3 (mixture of additive and dominant effects) the GBLUP uses only matrices $K_A$ and $K_D$. In scenario S8 the matrices $K_A$, $K_D$ and $K_{DD}$ are included. The only scenario that uses all matrices is thus S9. In the rest of the paper this model with a varying selection of kinship matrices will be referred to as GBLUP-optim.

### Deep neural network architecture

A sequential neural network architecture can be thought as an oriented graph. In the first layer, all nodes are connected directly to the network input and their output is fed to a second set of nodes called the second layer. Data proceed through the network in a sequential fashion until it reaches the last, output layer, and a value is then produced. The number of layers and the number of nodes in each layer are key aspects of a sequential neural network.

We evaluated several architectures, each built using a combination of the following components:

- *Dense layer*: standard neural network component, contains $d_i$ nodes, with *i* swiping the number of dense layers. Each node performs a linear combination of the input values. The result is either output directly or fed through a nonlinear activation function. We chose the ReLU function that outputs zero if fed a value lower than zero, or the value itself if fed a value above zero.
- *2D convolutional layer*: containing $c_i$ convolutional nodes, each performing a 2D convolution over a sliding window that swipes the full image.
- *Max pooling layer*: downsamples the input along its spatial dimensions (height and width) by taking the maximum value over a sliding window for each channel of the input.
- *Flatten layer*: used to rearrange data output from convolutional layers (where data has the shape of a 2D image, possibly multichannel) to the dense layers (where data need to be in the form of a one-dimensional vector).
- *Dropout layer*: only during training time, this layer randomly sets layer nodes to 0 with a fixed frequency drop rate ($D_{rate}$) at each step, which helps prevent overfitting. Inputs not set to 0 are scaled up by 1/(1 - $D_{rate}$) such that the sum over all inputs is unchanged.

A specific feature of deep learning is that the model hyperspace is huge, and it is therefore virtually impossible to explore it completely. We adopted a heuristic approach to progressively select the most promising options and parameter values, thereby incrementally building our architecture until the final model was eventually defined. We named our approach *greedy stepwise model selection*, since we took one hyperparameter at a time, optimized it and then move on to the next. This approach operates under the hypothesis of orthogonality between hyperparameters^31^, i.e. the order in which the hyperparameters are optimized is not relevant.

**Figure 3:**
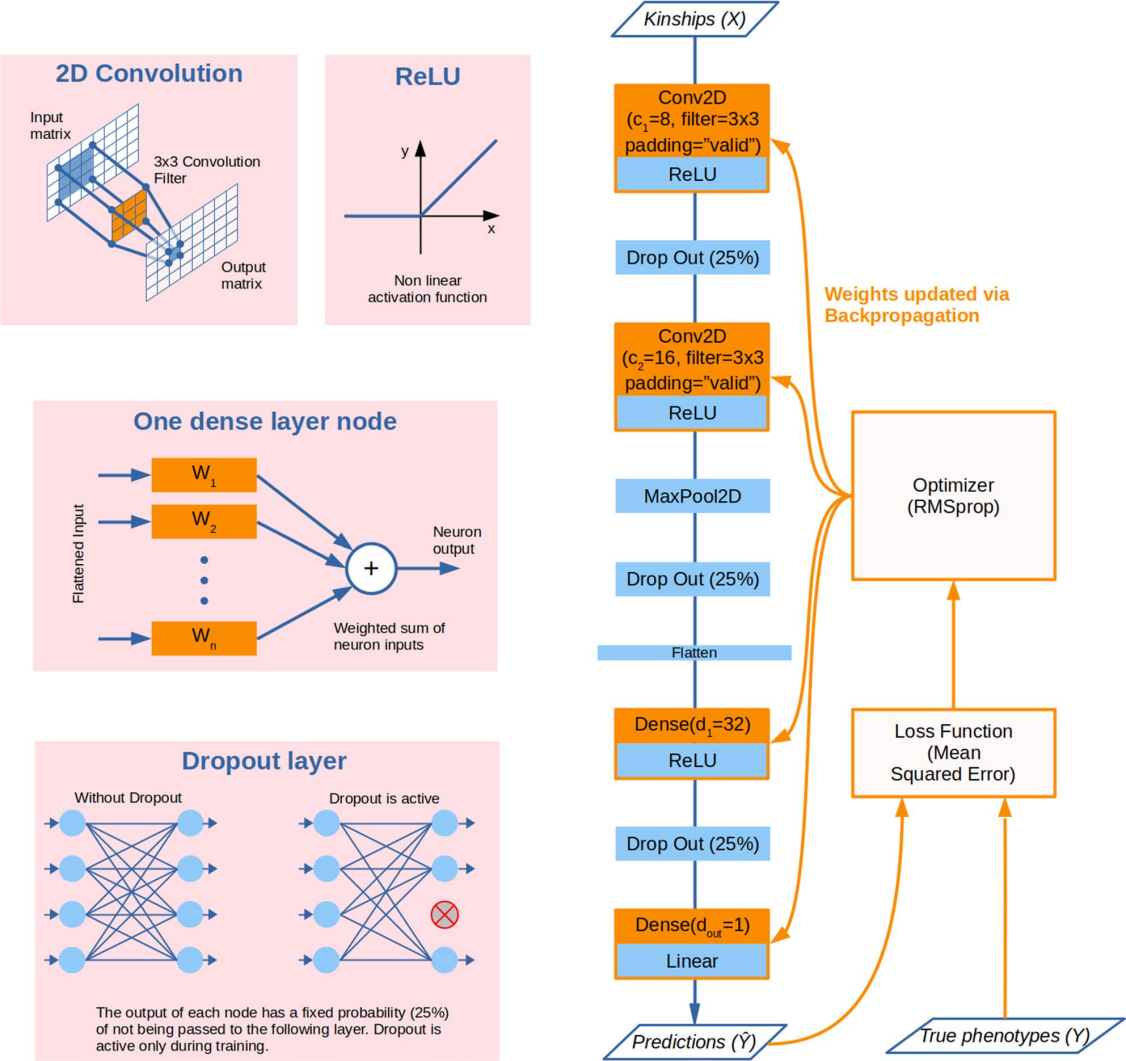
The base deep learning architecture used as a starting point for hyperparameter tuning. The kinships are inputted as a $n\ {\times }\ n\ {\times }\ 15$ polyhedron, where *n* is the number of samples for the training set and 15 is the number of kinship matrices used. Trainable layers are represented in orange. All layers with the exception of the last one use the ReLU activation function. The last layer always has a single node ($d_{out}=1$) and uses a linear activation function so to produce a continuous output value.

We started from a basic network with three layers (two convolutional layers plus one dense layer, besides the output layer) in addition to support layers (one dropout layer after each intermediate layer, one max pooling layer after the second CNN layer, one flatten layer to pass from CNN to dense layers), as reported in Fig. 3. We used Pearson’s correlation on the validation set as a metric to decide between different options. Each option was evaluated as the average of 10 replicates (resampling the training/validation split). All runs for model building were tested on the completely additive phenotype (S1) from Simulation 0. This was motivated by the impracticability in terms of time and computational resources of reoptimising each time all hyperparameters and the network architecture. The current state-of-the-art approach is to optimise a network developed for similar types of problems (e.g. ResNet50^32^, EfficientNet^33^) which is then retrained on the new data but not redesigned, an approach known as transfer learning.

The number of epochs was fixed to 300 after preliminary testing showed that it was enough to exhaust the training period (data not reported). We then tuned, in order: (1) the size of the filter (kernel) in convolutional layers, with tested values: $3\times 3$, $5\times 5$, and $7\times 7$; (2) the type of padding for convolutional layers, with tested values: “same” and “valid”; III) the network architecture in terms of number of nodes and layers, trying different combinations of convolutional and dense layers, up to six layers. See Supplementary Table S1 for the list of considered variants.

Once the general architecture was fixed we tested three regularization approaches to reduce the accuracy gap between training and test set and push the network to generalize better. First, we added L1 and L2 regularization layers to all learning nodes (i.e. convolutional and dense nodes), with tested values for $\lambda _1$ and $\lambda _2$ (penalty coefficients) in [0, 0,1, 0.01, 0.001, 0.001, 0.00001]. Second, regularization due to dropout was fine-tuned by trying out different values for the dropout rate $D_{rate}$: 0 (no dropping out of nodes), 0.10, 0.25 (baseline value), 0.40 and 0.50. Finally, we tested data augmentation by adding to the kinship polyhedron further copies of the data with random Gaussian noise injected to the kinship matrices: 1$\times$, 2$\times$ and 4$\times$ data augmentation were explored (i.e. 1, 2 or 4 copies of the data added). Injected noise was sampled from a normal distribution with null mean and standard deviation ranging from 0.01 to 0.3.

**Table 2:** Successive model-building steps.

Step	Hyperparameter	Selected value	Pearson accuracy on validation set	Progressive improvement	Improvement within the step
1	2D convolution kernel size	3 $\times$ 3	0.510	–	24.09
2	Padding	“Same”	0.514	0.78	4.70
3	Layers numbers and type	[32 64][64 32 16]	0.583	13.42	42.20
4	L1–L2 regularization	None	0.583	0.00	95.64
5	Drop rate	0.25	0.583	0.00	163.80
6	Data augmentation	$reps=1\times ;\ \sigma =0.1$	0.619	6.22	21.07

The starting network was a three-layer network. Each table row represents the optimization of a single hyperparameter, with the selected value and the obtained accuracy on the validation set. The last two columns report the percentage improvement in model performance (measured as Pearson accuracy) relative to the previous step or within each step (best vs. worst performance) [*reps*: data replicates for augmentation; $\sigma$: standard deviation of the Gaussian noise introduced in the data].

### Cross-validation scheme and performance metrics

Both GBLUP-based and DNN models were trained with a 80/20 validation set (hold-out) approach. At each training round a subset consisting of 20% of the available samples was randomly selected and used for validation. The remaining 80% of the data was used for training the model. To ensure numeric stability the full subsetting/training cycle was repeated ten times for each model-phenotype combination. Additionally, the whole process has been repeated three times, each time on a different set of the S1–S9 simulated phenotypes (Simulation 1, Simulation 2, Simulation 3).

We measured each model’s performance using three distinct metrics. All metrics were directly comparable across models, since all phenotypes were standardized to zero mean and unitary variance. Predictive ability was measured as the Pearson’s linear correlation between true and predicted phenotypes. This produced a score between plus one (perfect correlation) and minus one (perfect anticorrelation) with a value of zero representing absence of correlation.

The second selected metric is root mean squared error (RMSE), and is computed via the following formula:

$$\begin{aligned} RMSE = \sqrt{\frac{1}{n} \sum _{i}^{n} (y_i - {\hat{y}}_i)^2} \end{aligned}$$

where *y* is the set of all true phenotypes in the validation set; ${\hat{y}}$ is the set of predictions; and *n* is the number of samples. RMSE by definition is a positive number, but has no upper bound. RMSE is expressed on the same unit of measurement as the original data.

The third measured metric is normalized discounted cumulative gain (NDCG), a metric used to evaluate ranking quality that gives extra importance to the top-ranking samples^34^. NDCG works under the hypothesis that higher values for the phenotype are desirable. It is obtained in two steps. First the discounted cumulative gain (DCG) is computed:

$$\begin{aligned} DCG(y, {\hat{y}}) = \sum _{i=1}^{n} \frac{{\hat{y}}_{rank(y_i)}}{log_2(i+1)} \end{aligned}$$

The numerator swipes the ${\hat{y}}$ array of predictions, but follows the order imposed by the y array of true values. Thus, when $i=1$ the numerator will be equal to the prediction of the true top ranking sample. The denominator grows indefinitely, thus the first few terms of the summation give the main contribution and the later terms, being divided by bigger and bigger denominators, contribute less. Intuitively, if top ranking (i.e. good, higher values) samples end up in congruous top-ranking positions in the predictions the total DCG will be higher. Conversely, if bottom ranking (i.e. bad, lower values) samples end up in top-ranking positions in the prediction set the total DCG will be lower. DCG could be used by itself, but as a metric it has some limitations. Its value can be either positive or negative (since it depends on the phenotypes) and there is no upper limit. Moreover, it depends on the size of the validation set. It is therefore preferable to normalize DCG and compute NDCG:

$$\begin{aligned} \textit{NDCG}(y, {\hat{y}}) = \frac{DCG(y, {\hat{y}})}{DCG(y, y)} \end{aligned}$$

The denominator, sometimes called Ideal DCG, is the value obtained with perfect predictions, i.e. when true and predicted values are identical. As a metric NDCG is bound to one as its upper limit. The closer to one, the more congruous the top-samples ranking.

### Software implementations

Simulated phenotypes were generated using the *sim.phe* function from *SimPhe* R package^35^ version 0.2.0. Kinship matrices were calculated with the R package *sommer*^36^ version 4.1.3.

GBLUP models were built and trained using the GROAN R package^37^, released together with this work as version 1.3 with NDCG support and multi-matrix GBLUP models. Models from Eq. (2) were implemented using the *phenoregressor.BGLR.multikinships* function with Gibbs-sampling options *nIter = 10000* and *burnIn = 500*.

Neural network models were built and trained using the Keras Python library (https://keras.io/) version 2.8. Dense layers were implemented using Keras *layers.dense* function with parameter *activation = ‘relu’*, with the exception of the very last layer that has only one node and uses *activation = ‘linear’*. 2D convolution layers were implemented using Keras *layers.Conv2D* function with parameters *activation = ‘relu’, padding = ‘same’, kernel_size = (3,3)*. Max pooling layers were implemented using *keras.MaxPooling2D* function with parameter *pool_size = (2,2)*. Flatten layers were implemented with the *keras.flatten* function. Dropout layers were implemented using *keras.Dropout* function with parameter *rate = 0.25*. All DNN models were trained using the RMSprop optimizer implemented via *keras.optimizers.RMSprop* function, with parameters *learn_rate=0.001* and *loss = MeanSquaredError()*.

All scripts (R scripts for simulated phenotype generation and GBLUP models, Python notebooks for neural network models) are released via the public Github repository filippob/paper_deep_learning_vs_gblup (see details below).

## Results

### Genotypes and simulated phenotypes

The 26,503 SNP marker data from the 1033 Holstein Friesian samples had average MAF = $0.2785\pm 0.138$, with $36.5\%$ average heterozygosity. From principal components analysis (PCA), the plot of the first two components explained $\sim 70\%$ of the genetic variability and did not show any clustering or population substructure (Supplementary Fig. S1). When filtering for MAF $>1\%$, MAF $>5\%$ and MAF in 1–5% (rare variants), 26,202, 24,923 and 1279 SNPs were left to build the kinship matrices, respectively.

We simulated nine different phenotypic scenarios ($S1, \dots , S9$), each representing a different trait architecture and mixing additive, dominant and epistatic effects. The whole generation process was repeated four times (Simulation 0, 1, 2, and 3) leading to a total of 36 sets. Data from Simulation 0–S1 was used for tuning the network architecture. The remaining three simulation runs where all used for testing purposes.

Given the polygenic nature of the simulated phenotypes, the final distributions of traits are all expected to tend to normality as per the central limit theorem^38^. In addition, at each iteration (either changing phenotype scenario or simulation run) the set of involved QTN was randomly resampled. This completely broke the link between different phenotypes, which are thus expected to be uncorrelated. Both hypotheses are confirmed in Supplementary Fig. 2, which reports the distribution plots, both univariate and paired, for the 9 phenotypes (S1–S9) from Simulation 0.

### Model building

**Figure 4:**
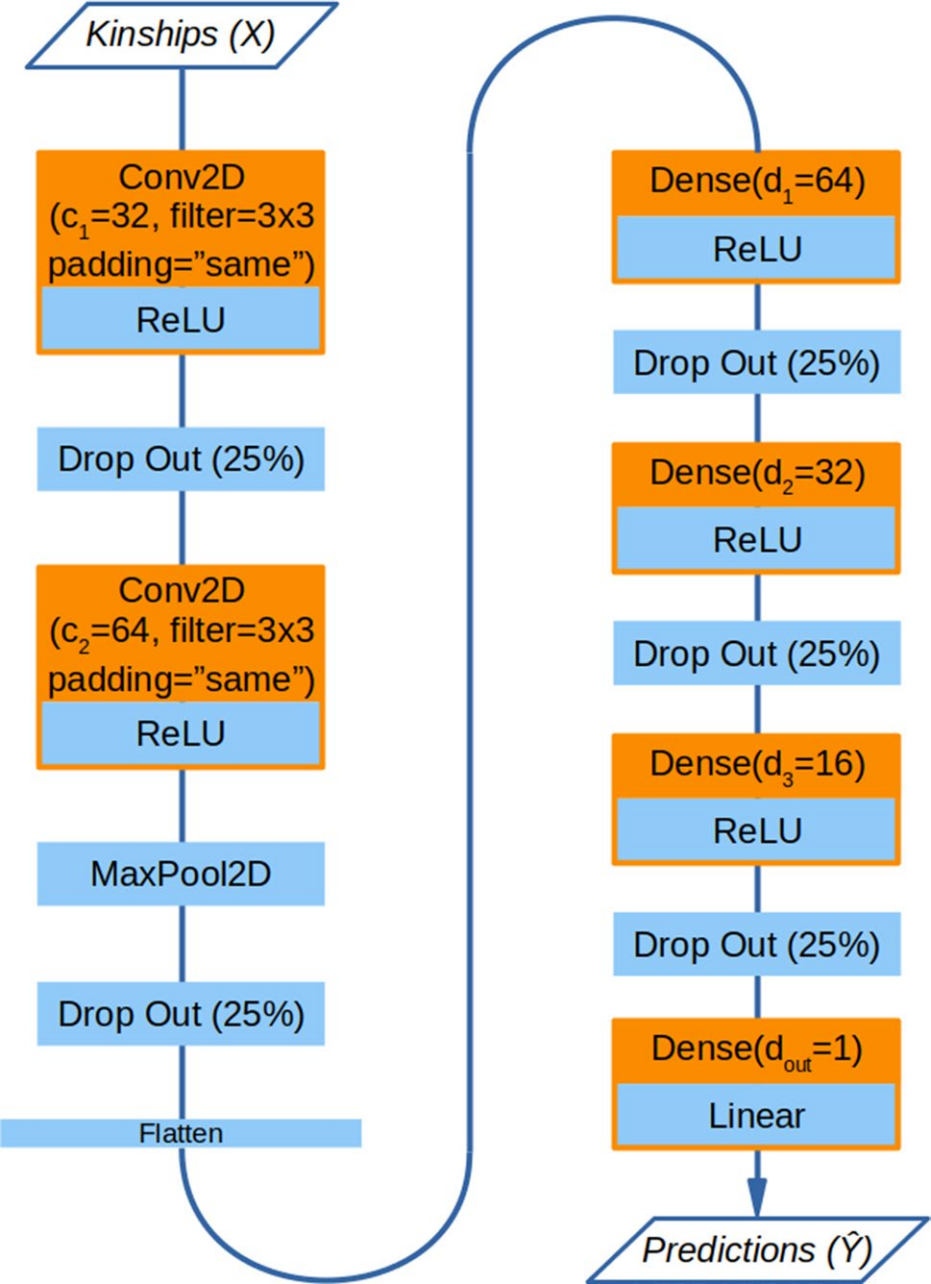
Final deep learning architecture selected after hyperparameter tuning. Trainable layers are represented in orange. All layers with the exception of the last one use the ReLU activation function. The last layer always has a single node ($d_{out}=1$) and uses a linear activation function so to produce a continuous output value.

The final architecture (Fig. 4) was obtained from a base network via step-wise optimization of the considered hyperparameters (*greedy stepwise model selection*). The selected hyperparameters were: (1) the size of the filter (kernel) in convolutional layers: 3 $\times$ 3 gave the best results (accuracy = 0.510) over 5 $\times$ 5 and 7 $\times$ 7 filters; (2) the type of padding for convolutional layers: “same” padding (accuracy = 0.579) was selected over “valid” padding; (3) the network architecture the best results were obtained using 2 convolutional layers followed by 3 dense layers with [32, 64] and [64, 32, 16] nodes respectively (accuracy = 0.59).

At this point the architecture was fixed, but still resulted in a substantial gap between accuracy in the training and test sets ($\sim 0.95$ vs $\sim 0.59$) due to the DNN model overfitting the data. To tackle these discrepancies we tested three regularization approaches. First we tested the effect of adding L1 and L2 regularization layers to all trainable nodes (i.e. convolutional and dense nodes). We tested $\lambda _1$ and $\lambda _2$ (penalty coefficients) with values in [0, 0, 1, 0.01, 0.001, 0.001, 0.00001]. However, none of the L1–L2 regularized models gave better results than the model without L1–L2 penalties and were thus discarded. Regularization due to dropout was fine-tuned by trying out different values for the dropout rate $D_{rate}$. However no improvement was obtained over the baseline value of 0.25 for $D_{rate}$, which was therefore retained as optimal. Finally, data augmentation with noise improved the performance of the DNN model, with the best results obtained with 1$\times$ augmentation and $0.1\sigma$ noise (accuracy = 0.619).

The final model was then used for all subsequent analyses. Table 2 reports the subsequent steps of model tuning, with the relative increase in performance. The detailed summary of all the steps involved in model building is reported in Supplementary Table S1.

### Model evaluation

**Figure 5:**
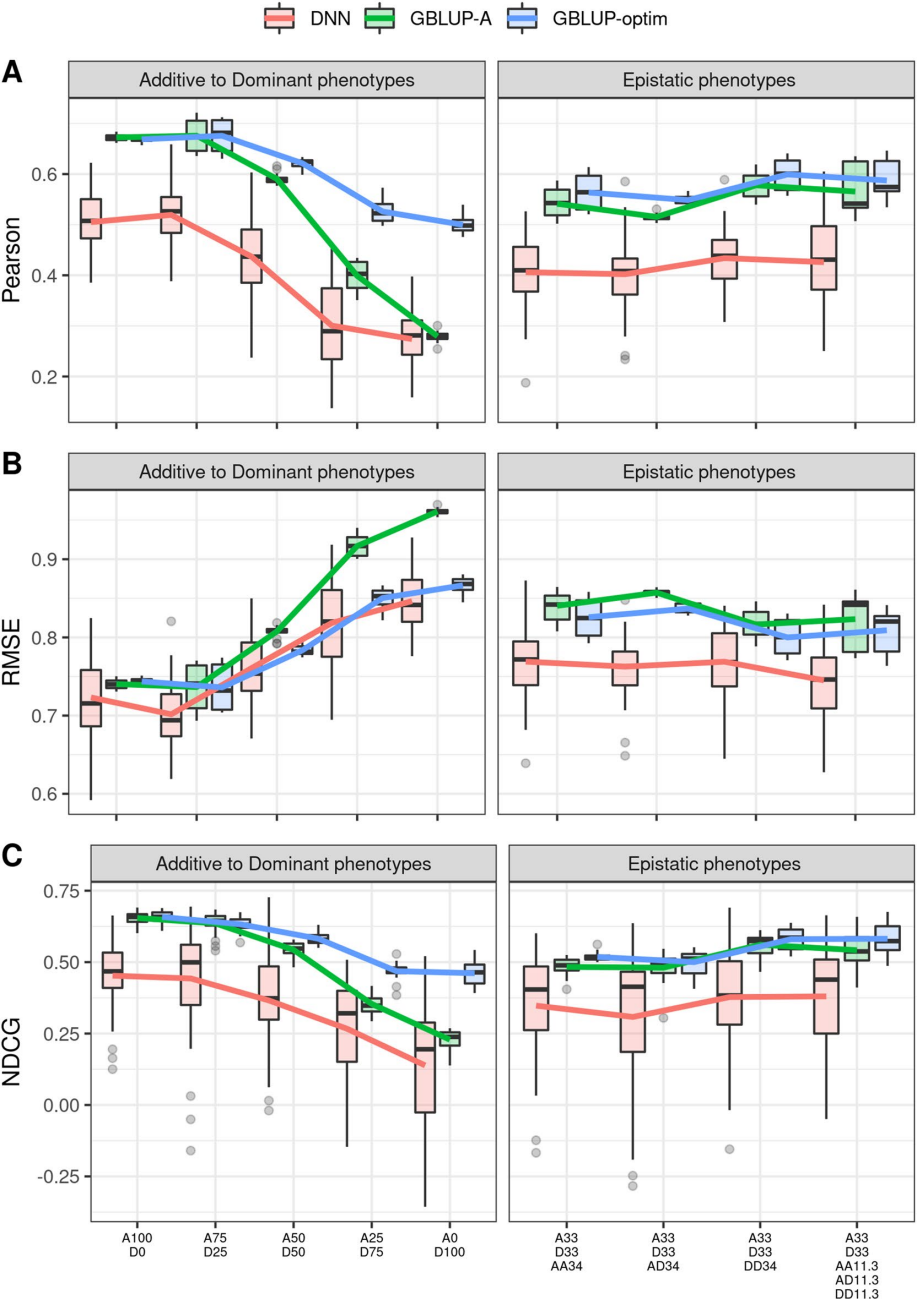
Predictive ability of deep learning and GBLUP models for the prediction of phenotype with different proportions of additive, dominance and epistatic effects. Performance is expressed in terms of: (**A**) Pearson’s correlation; (**B**) Root Mean Squared Error; (**C**) Normalized Cumulative Discounted Gain. Boxplots show the median and distribution of predictive ability. Solid lines represent the average predictive ability under the different scenarios.

The core results from the GBLUP and DNN models are reported graphically in Fig. 5 and with more details in Supplementary Table S2. For GBLUP, two models are presented, GBLUP-A GBLUP-optim. The first one always uses a single additive kinship matrix, while GBLUP-optim uses a variable number of kinship matrices (up to five) depending on the combination of genetic effects determining the phenotype (additivity, dominance, epistasis). DNN uses the architecture optimized for the completely additive phenotype from Simulation 0. All models are tested on three different sets of synthetic phenotypes (Simulation 1, 2, 3) using a 80/20 validation split, repeated 10 times for numerical stability.

In general GBLUP models yielded clearly higher predictive ability in terms of Pearson’s correlation between observed and predictive phenotypes, and of NDCG. For the fully additive phenotypes (Scenario S1, A100D0) GBLUP-A and GBLUP-optim are equivalent (only using additive kinship) and resulted in very similar values for Pearson’s correlation (0.676 and 0.669, respectively) and NDCG (0.658, 0.655). DNN obtained a correlation of 0.505 and an NDCG of 0.452, but showed better results in terms of RMSE (DNN = 0.723, GBLUP-A = 0.739, GBLUP-optim = 0.744). This pattern applied consistently to all phenotypes, along the additivity, dominance and epistasis gradients, with DNN’s RMSE 1–12% lower than GBLUP-A’s RMSE and 1–9% lower than GBLUP-optim’s RMSE depending on the scenario.

Additive and GBLUP-optim models gave comparable performances for phenotypes with predominantly additive components. The performance of the two models diverged when the dominant component determined $\ge$ 50% of the phenotype: in these scenarios, the predictive ability of additive GBLUP models sharply declined, while GBLUP-optim models coped better with the increasing non-linearity of the data. DNN models showed a trend very similar to GBLUP-optim models but with a much less dramatic reduction of predictive ability with increasing dominance. In the case of purely dominant phenotypes GBLUP-A and DNN had very similar predictive ability in terms of Pearson’s correlation and NDCG, with DNN maintaining a better RMSE than both GBLUP models.

In terms of Pearson correlations, all results for the DNN models but the fully dominant phenotype (D100) were significantly different from GBLUP-A results (p value $<0.05$ from analysis of variance), while for GBLUP-optim this was the case only for two phenotypes (A0–D100, A25–D75: Fig. 5A). In terms of RMSE, all DNN results but for the fully additive phenotype (A100–D0) were significantly different from GBLUP-A results, while for GBLUP-optim this was the case only for four phenotypes (A0–D100, A25–D75, A33–D33–AA34, A50–D50: Fig. 5B). In terms of NDCG, all DNN results were significantly different from GBLUP-A results, while for GBLUP-optim this was the case only for two phenotypes (A0–D100, A25–D75: Fig. 5C). All p-values are reported in Supplementary Table S3.

In most cases, GBLUP models showed lower variability than DNN models, indicating less sensitivity to the specific subset of samples used for training. In summary, the presence of dominant components in the phenotypes negatively influenced the performance of all models, while the specific type of epistasis had a less pronounced effect.

## Discussion

In this study, we presented the results from an innovative modeling of whole genome predictions using convolutional neural networks on multiple stacked kinship matrices. The deep learning model was fitted to a number of simulated phenotypes under different combinations of additive, dominant and epistatic genetic effects, and performances were compared to those of GBLUP models that accommodated different numbers of kinship matrices. The proposed methodology attempts to reconcile two very successful paradigms: on one hand neural networks—CNN in particular—applied to the analysis of image data and able to solve traditionally hard to tackle problems; on the other hand GBLUP, successfully applied to solve $p>> n$ problems which are typical of ’omics predictions. However, GBLUP is known to suffer when the phenotype is not linear. Our approach shows a GBLUP-inspired possible solution for the problem of inputting wide data (e.g. thousands of SNPs) into a CNN. Overall, results were mixed and depended on the metric of choice. The DNN model gave smaller RMSE and on average showed lower bias, in particular under non-linear scenarios. GBLUP models performed better in terms of correlation, NDCG and variance. Hereby we discuss some relevant aspects of results.

### Comparison to the state of the art

The problem of whole-genome predictions of continuous phenotypes proves to be particularly hard to tackle for deep learning, given that state-of-the-art penalized regression models already achieve nearly optimal-predictive ability. An interesting example was provided by Gill et al.^39^, who showed that other machine learning methods (i.e. random forest and XGBoost) outperformed deep learning on a soybean dataset, with the extra benefit of model interpretability and data insights. One hidden cost in deep learning approaches is often the training time, since strategies may include the optimization of network architecture and hyperparameters on each training iteration^11^. In terms of architectures, convolutional networks (CNN) seem in general more promising than dense networks^12^, although, a really convincing modeling of whole-genome predictions with DNN has not been found yet.

A recent study^40^ has proposed the principle of shortcut learning to explain why deep learning does not outperform penalized regression methods. Briefly, it is theorized that neural networks tend to base their predictions on overall genetic relatedness rather than on the effects of specific markers with, say, epistatic effects. In other words, starting from SNP data, the network was able to model internally something equivalent to a kinship matrix, which only accounted for additivity aspects and lost marker-specific information (e.g. epistatic effects). If confirmed, this emerging property would represent a limit in the use of neural networks as whole genome regressor, at least starting from raw SNP data.

Among the latest developments, interesting approaches are based on the idea that genomic data can be converted into image-like data to be fed to CNN deep learning models^41,42^: these ideas seem promising, but need to be further explored to assess their merit in the context of whole-genome predictions. Our work follows in this path and investigates data representation as a possible core component of the training process. At the best of our knowledge no other work has yet used kinship matrices (single or combined) as input data to the network. While our solution is not yet “competitive” with nearly-optimal GBLUP, it takes an interesting and promising step in the right direction and we believe that any kind of application of CNN to whole-genome predictions needs to take into account the problem of data representation.

### Rationale on data representation

It is well known that from the mathematical standpoint the problem of whole-genome regression is ill posed, with the number of independent variables (i.e. the SNPs) usually many orders of magnitude greater than the number of available samples. Many regularization techniques have been proposed to be able to regress directly on the SNP data, e.g. ridge regression, Bayesian Lasso and the whole Bayesian alphabet^43^. Genomic BLUP uses a different parameterization of the problem to reduce the dimensionality of the genomic data^30^. Under the assumption that additive effects can be captured by an aptly constructed kinship matrix, GBLUP faces the computationally easier problem of using a square (samples $\times$ samples) covariance matrix. Moreover, other types of matrices can be built and added to the model to intercept non additive aspects of the phenotype^44^.

A similar intuition can be applied to neural network models. DNN models can automatically learn features from the input data, thus avoiding handcrafted feature extraction^45^. Yet, SNP matrices (samples $\times$ markers) are cumbersome as input data, with a single data point (i.e. all the SNPs referring to the same sample) consisting often of tens of thousands SNPs. This dimensionality is simply too much for a standard dense neural network, where each node of the first layer should train a weight for each input SNP, leading to dramatic overparameterization (e.g. 50 k SNP data and a dense layer with 64 nodes would result in more than three millions parameters to train only the first layer). An interesting aspect of DNN is that it automatically learns features from the input data^45^. Kinship matrices are thus an interesting candidate for dimensionality reduction also for deep learning models. Collating different matrices allows in a single pass to input different types of effects (additive, dominant, epistatic) and filtering criteria (e.g. MAF thresholds) to the network. Moreover, this approach aims to leverage the capability of convolutional neural networks to extract spatial correlations, i.e. patterns in the similarity between samples and different effects. In our chosen data representation we ordered samples hierarchically (based on the additive kinship matrix) to highlight patterns of similarity in the 2D data matrix fed to the first convolutional layer. Additionally, this data representation can easily be extended to include other strategies of kinship computation. In this work, we haven’t tested the use of a set of kinship matrix different from the 15 matrices obtained from the 5 types of genetic relationships ($K_A$, $K_D$, $K_{AA}$, $K_{AD}$, $K_{DD}$) and the three MAF filters presented here. A larger number of kinship matrices could be stacked together, or only those matrices as used in GBLUP-optim could be selected; this latter approach would result in a very thin polyhedron (e.g. one with only one or very few kinships) and will thus present a very elongated slice to the neural network, expected to yield worse model performance. These aspects, though, remain to be further explored.

### The train-validation performance gap

In this work we considered three different regularization techniques, namely L1/L2 regularization, dropout layers and data augmentation with noise. While different in implementation, all these approaches share the common goal of stopping the neural network from overfitting the training set. This target has been only partially achieved. Supplementary Fig. S3 shows one training trajectory, reported as a representative example of very similar behavior observed in all experiments. After the initial setup phase the accuracy on the training set often reached very high values, e.g. obtaining a Pearson’s correlation of 0.98, way above the theoretical maximum value of 0.7 imposed by heritability. The performances measured on the validation set were always lower, and the gap between train and validation sets remained. This is a sign of overfitting. In ideal conditions the network would be able to correctly generalize its learning and the performances on training and validation should be similar^46^.

In this framework it is interesting to observe that different regularization techniques resulted in widely different effects. L1/L2 regularization not only did not help, but worsened the overall performances, so much that we removed it from the final architecture. On the contrary, both the dropout layers and noisy-data augmentation improved the model accuracy by 34.9% and 6.2% respectively (see Supplementary Table S1). None of these techniques was however capable of completely closing the training-validation gap. It is thus expected that the use of novel regularization techniques could further improve the network performances.

A collateral effect of the training-validation gap is that it becomes unnecessary to optimize the learning rate, a hyperparameter commonly considered of importance in neural network training. The choice of a coarser or finer learning rate imposes the “step” used by the optimizer to explore the training space. Given that our networks were already overfitting, no improvement could have come from fitting the training set even better.

### Model space exploration

Neural networks model space is very wide and an exhaustive test of all possible combinations of hyperparameters quickly becomes impossible. In this work, we selected a stepwise optimization approach, optimizing one hyperparameter at a time and then keeping the temporary best configuration for the next steps. This approach led to a 21.4% accuracy increase (Pearson correlation) from the initial to the final network architecture (see Table 2) and defined the DNN architecture that we then applied to all phenotypes in all simulation rounds.

The described process could be partly automated using training assistance tools like Talos^47^, which help explore the grid of trainable parameters, or fully automated using machine learning methods^11,48^. This latter approach (ML) bears the promise of potentially interesting developments. Our network shows a clear drop in performance when moving from the phenotype used for architecture optimization (Simulation 0) to other phenotypes (Simulations 1, 2, 3). With reference to purely additive phenotypes to avoid confounding factors, the network resulted in RMSE 0.647 and Pearson’s correlation 0.619 on Simulation 0, and in RMSE 0.725 and Pearson’s correlation 0.503 as the average of Simulation 1, 2, and 3. An automated tool for hyperparameter optimization could in theory ensure performances on all phenotypes on par to what is obtained on Simulation 0. This would however widen the disparity in computation times even further. For reference, GBLUP training times ranged from 18 seconds in the best case scenario ($K_A$ kinship matrix) to 105 seconds for the worst case scenario ($K_A$, $K_D$, $K_{AA}$, $K_{AD}$ and $K_{DD}$ matrices) on a desktop workstation (AMD Ryzen 9 3900X 12-Core Processor). For comparison, a single 300-epoch training cycle of the DNN model required on average little more than 8 minutes on a Google Colab environment running a Tesla T4 GPU. Optimizing the hyperparameter selection at each training cycle would multiply the required time by the number of combinations tried, which are tens at minimum and hundreds in a realistic scenario (as already noted, for instance, by Zingaretti et al.^11^). The projected training time would thus easily amount to several hours, a very wide gap when compared to the minute and a half of the GBLUP worst case scenario.

### Neural networks privilege additive phenotypes

The simulated phenotypes used for this study comprise a wide array of genetic architectures, some of which are very well suited to be predicted using GBLUP. This becomes evident in the case of purely additive phenotypes (A100–D0), where GBLUP-A achieved a predictive ability of 0.67, very close to the limit imposed by heritability ($h^2 = 0.7$). As a consequence any type of improvement, even one reaching the theoretical limit, could only be marginal when compared to GBLUP’s already nearly-optimal accuracy. Real world data would pose additional difficulties, e.g. genotype x environment interactions, incomplete coverage of the involved QTNs, and the combined effect of the genes in the background (the so-called missing heritability problem)^49,50^. Avoiding these confounding factors by using simulations, this study focuses on the effects of different genetic architectures on the studied phenotypes.

The most evident trend is detected on the gradient from purely additive (A100–D0) to purely dominant (A0–D100) phenotypes, where GBLUP-A’s predictive ability drops from the 0.67 cited above to 0.28. Setting aside the biological plausibility (or absence thereof) of a purely dominant complex quantitative phenotype, this result highlights a known GBLUP limitation. Dominant effects introduce a nonlinear relationship between genome and phenotype which is captured with difficulty by a linear model. Even GBLUP-optim, which uses a kinship matrix explicitly designed to intercept the dominant components of the phenotype, sees its performance drop when moving away from purely additive phenotypes. The DNN closes the gap with GBLUP-A’s predictive ability only in case of purely dominant phenotypes, and never for GBLUP-optim. Conversely, the DNN’s RMSE is constantly lower (i.e. better) than both GBLUP models.

Most interestingly, the DNN model follows the same degradation pattern seen for GBLUP, with a predictive ability that almost halves when moving from a purely additive to a purely dominant phenotype. Similar degradation patterns for GBLUP and for DNN models have already been reported^28,51^. This is circumstantial evidence that the neural network is modeling the relationship between genotypes and phenotypes as linear, thus suffering the same limitations of GBLUP. As a matter of fact, if the DNN were able to disengage from an additive internal modelization it would have achieved always the same performances over the additive-dominant gradient. On the contrary, its degrading performances denounce a marked tendency toward linear phenotypes.

This hypothesis is corroborated by the results obtained on phenotypes with epistatic components. The major trend is a general flatness on predictive ability, RMSE and NDCG. No model was affected by a particular type of epistatic effect or combination thereof, reporting roughly the same values for all metrics. When compared to the additive–dominant gradient, all models resulted in performances similar to what is obtained with A50-D50, with DNN’s behavior again very similar to what observed for GBLUP. It must be noted that for real world data there is evidence that the summation of many (small) epistatic effects can bring to the emergence of additive genetic variance^52^. Thus, models geared for linear phenotypes could still be well suited for the task of genomic predictions even in presence of epistasis.

### Metrics: how prediction accuracy is measured

From our results we noticed that GBLUP models consistently showed higher prediction accuracy expressed as the correlation between observed and predicted phenotypes or using NDCG, while DNN models were consistently better in terms of lower RMSE. This held across all the phenotypic scenarios that were simulated. This contrasting behavior of GBLUP vs deep learning models has been already observed in several studies on whole-genome predictions in animals and plants^53^.

The difference in prediction accuracy measured with different metrics may not be irrelevant. In the case of continuous target variables, predicted and observed values may be highly correlated, yet their absolute difference may be large, with relevant practical implications. This is illustrated by the GoogleFlu predictions of influenza-like cases in 2013 in the United States which, in spite of highly correlated trends, were predicted to be the double of the actual cases^54^.

It is not clear why GBLUP and DNN models behave differently when the accuracy of predictions is measured by linear correlations or RMSE. A tentative explanation could come from the fact that the neural network optimizer aims to minimize its loss function, MSE, which is strictly linked to RMSE. We tested this hypothesis using the *cosine similarity*, a different loss function present in the Keras library that aims to minimize correlation between predictions and true phenotypes. Unfortunately it performed very poorly (data not reported) and this option was soon discarded.

A different, theoretical explanation for the observed differences between metrics may be related to the bias of predictions obtained from the different models. GBLUP is a linear mixed model, characterized by low variance and relatively high bias. On the contrary, deep learning is capable of accommodating highly non-linear relationships in the data, and therefore is expected to show high variability but low bias of results^55^. From the entire set of individual predictions obtained for all phenotypes and replicates (Simulations 1, 2 and 3) we calculated the bias of predictions. Based on the decomposition of the mean square error in the bias, variance and irreducible error components^55^, we calculated the bias term as:

$$\begin{aligned} bias \left( {\hat{f}}(x) \right) = E \left[ {\hat{f}}(x) \right] - f(x) \end{aligned}$$

**Figure 6:**
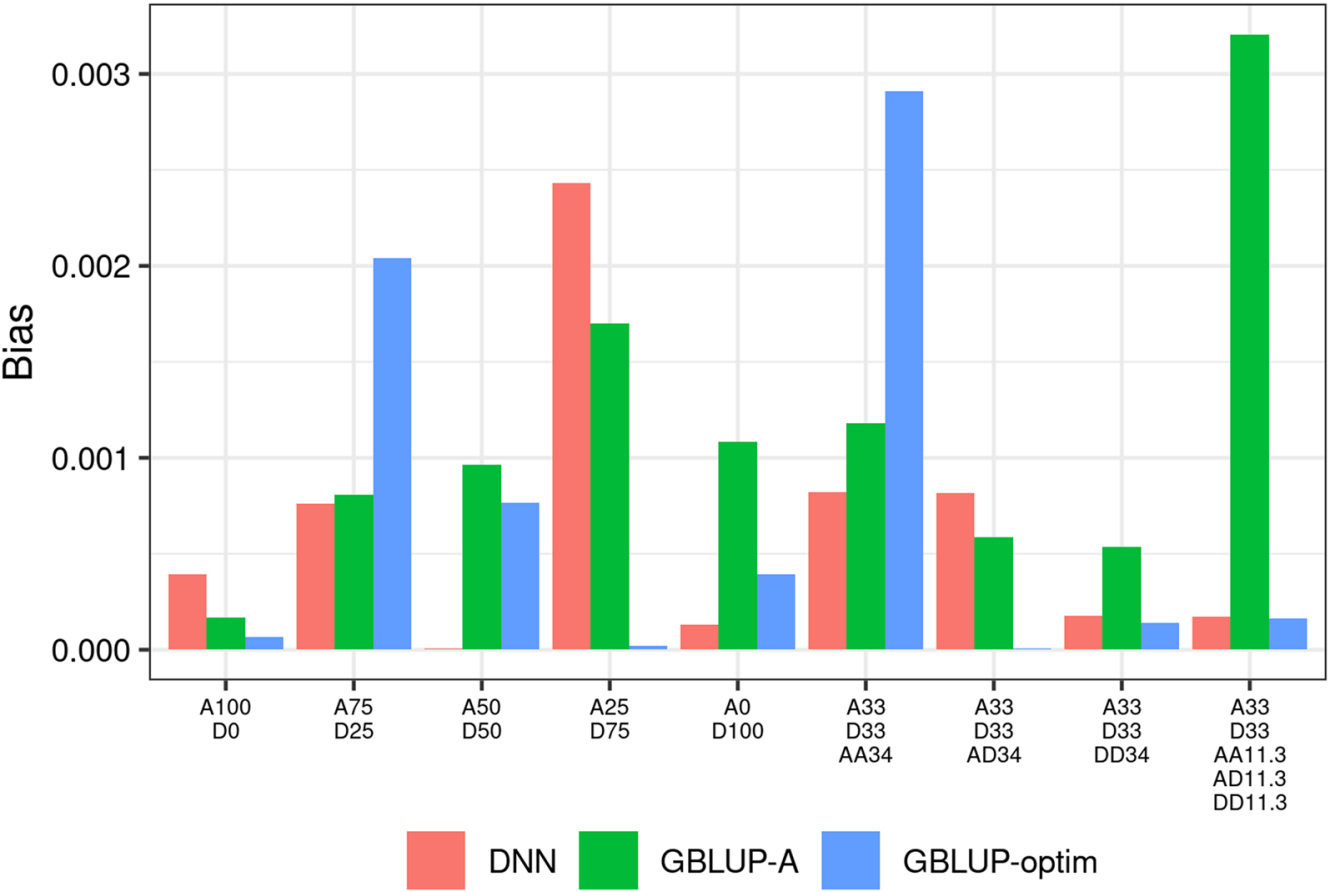
Bias of predictions for the different models across the range of simulated phenotypes (from purely additive, to purely dominant, to different flavors of epistasis). *DL* deep learning, *GBLUP-A* GBLUP with the additive relationship matrix only, *GBLUP-optim* GBLUP with multiple relationship matrices corresponding to the nature of the phenotype.

The bias for the different phenotypes and models is reported in Fig. 6. The results are not always consistent, however they show that (1) for GBLUP-A, the bias tends to increase with the phenotype complexity: it has low bias when the phenotype is purely additive, and large bias when the phenotype deviates progressively from the additive model; (2) for GBLUP-optim, the bias more or less decreases with phenotype complexity, in agreement with its good predictive performance: multiple kinship matrices appear to be able to accommodate non-additive and non-linear genetic effects, at the expense of poorer performance with simpler phenotypes; (3) DNN has a more erratic behavior, with bias generally lower than GBLUP-A (6 times out of 9) but not often lower than GBLUP-optim.

### Conclusions and future works

An effective solution to using DNN models for whole-genome predictions has not yet been found. Some current approaches focus on data representation, for instance treating genomic data as image-like data. Our work fits in this area and is the first to present the use of stacked kinship matrices fed to a CNN architecture. Results are not fully convincing though, with DNN models performing worse, similar or slightly better than GBLUP models depending on the phenotype and on the metric used for model evaluation, for a fraction of the cost. Even in the presence of complex genetic architectures, for example when including dominant components which introduce non-linear relationships between the genotype and the phenotype, the proposed DNN implementation fails to outcompete the GBLUP benchmark.

The research topic is however far from exhausted, and our results may point to one of the aspects to be considered for the development of effective DNN models for whole-genome predictions. Improvements can come from either incremental modifications (e.g. optimal number of nodes and layers, better regularization techniques, improved optimizers) or from radical shifts (e.g. different data representations, different loss and activation functions, different network architectures) that could allow for better predictions in cases difficult to tackle for traditional models.

## Supplementary Information

Supplementary Figure 1.

Supplementary Figure 2.

Supplementary Figure 3.

Supplementary Table 1.

Supplementary Table 2.

Supplementary Table 3.

## Data Availability

All data used in this article are publicly available. Genotypic data were obtained from a previously published article, phenotypic data have been simulated. Hence, no biological sampling nor handling of animals whatsoever have been used in this study, and no ethical statement is necessary. Original genotypes where retrieved from the public repository https://zenodo.org/record/4937628#.YfU7x1tKhhF. We have uploaded ready-to-use genotype data and simulated phenotypes, together with the scripts used to run the GBLUP and deep learning models, to the Github public repository https://github.com/filippob/paper_deep_learning_vs_gblup.
