# Supplementary material for: Stacked kinship CNN vs. GBLUP for genomic predictions of additive and complex continuous phenotypes
Source: Sci Rep. 2022 Nov 18;12:19889. doi: 10.1038/s41598-022-24405-0 (PMC9674857; doi:10.1038/s41598-022-24405-0)

Stacked kinship CNN vs. GBLUP for genomic predictions of additive and complex continuous phenotypes

Nelson Nazzicari[1,+] and Filippo Biscarini[2,+*]

[1]CREA: Council for Agricultural Research and Analysis of Agricultural Economics, Research Centre for Animal Production and Aquaculture, Viale Piacenza, 29 - 26900 Lodi
[2]CNR: National Research Council, Institute of Agricultural Biology and Biotechnology, Via Bassini 15, 20133 Milan, Italy
*filippo.biscarini@cnr.it
[+]these authors contributed equally to this work

Supplementary Figure S1: plot of the first two principal components (PC1, PC2) from the principal component analysis (PCA) of the 26,503 SNP genotypes from 1,033 Holstein-Friesian cows used in the study. Between brackets, the percentage of the variability explained by the two components.

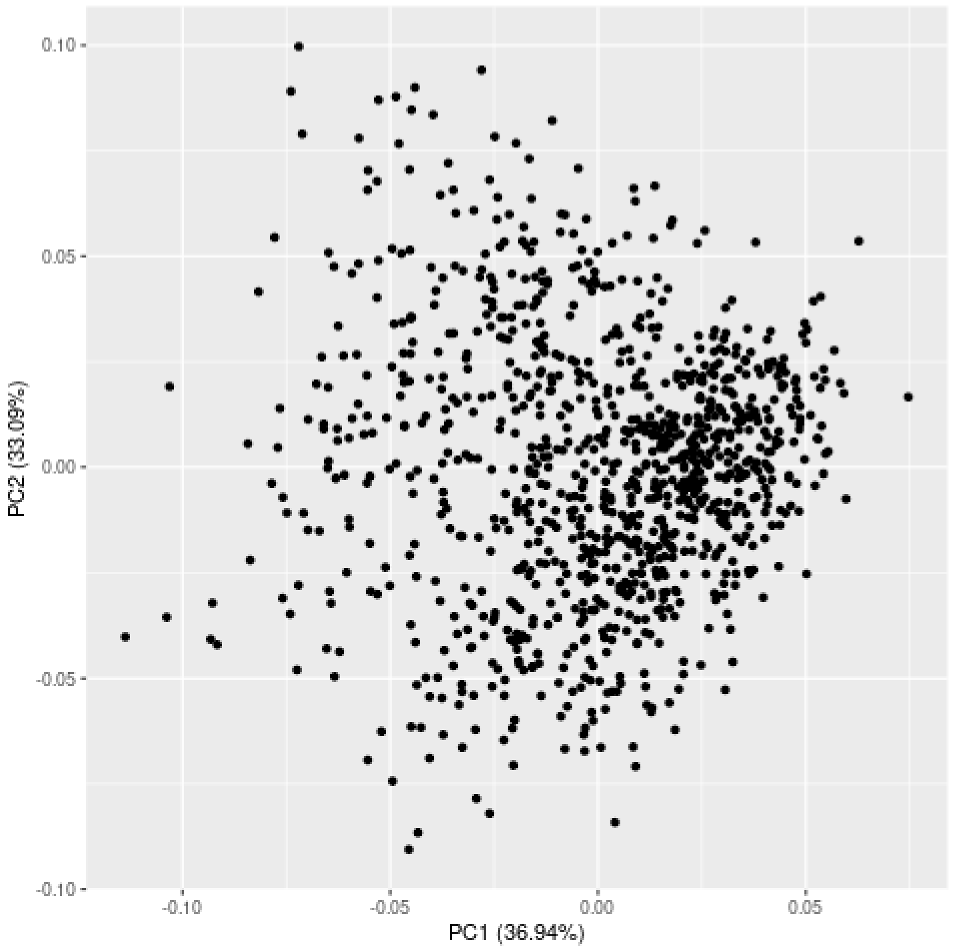

Supplement: Supplementary file 1 — Supplementary Figure 1. [file 41598_2022_24405_MOESM1_ESM.pdf]
