# Supplementary material for: Stacked kinship CNN vs. GBLUP for genomic predictions of additive and complex continuous phenotypes
Source: Sci Rep. 2022 Nov 18;12:19889. doi: 10.1038/s41598-022-24405-0 (PMC9674857; doi:10.1038/s41598-022-24405-0)

Stacked kinship CNN vs. GBLUP for genomic predictions of additive and complex continuous phenotypes

Nelson Nazzicari[1,+] and Filippo Biscarini[2,+*]

[1]CREA: Council for Agricultural Research and Analysis of Agricultural Economics, Research Centre for Animal Production and Aquaculture, Viale Piacenza, 29 - 26900 Lodi
[2]CNR: National Research Council, Institute of Agricultural Biology and Biotechnology, Via Bassini 15, 20133 Milan, Italy
*filippo.biscarini@cnr.it
+these authors contributed equally to this work

Supplementary Figure S2: distribution plots, both univariate and paired, for the phenotypes of Simulation 0 (S1-S9).

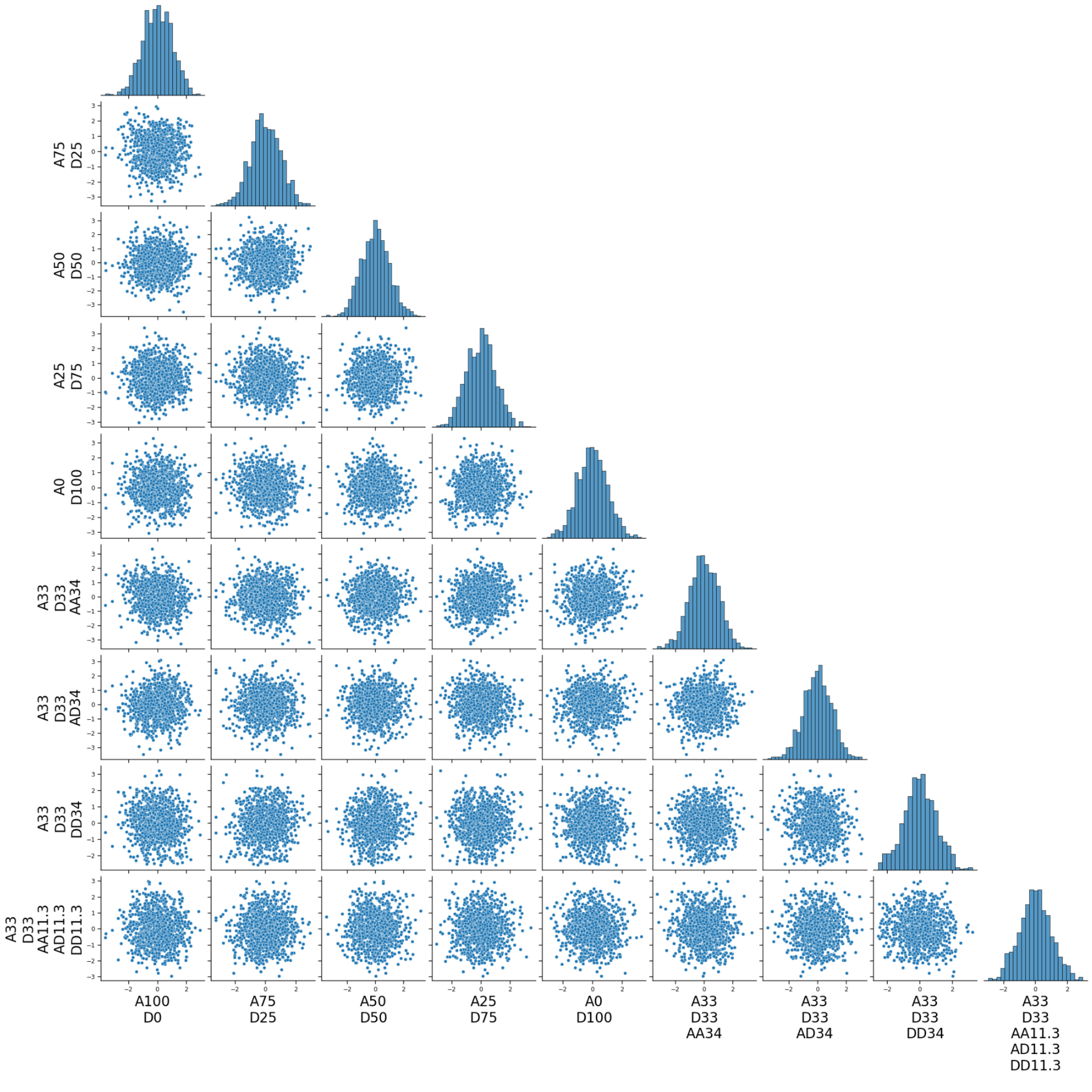

Supplement: Supplementary file 2 — Supplementary Figure 2. [file 41598_2022_24405_MOESM2_ESM.pdf]
