# Supplementary material for: Stacked kinship CNN vs. GBLUP for genomic predictions of additive and complex continuous phenotypes
Source: Sci Rep. 2022 Nov 18;12:19889. doi: 10.1038/s41598-022-24405-0 (PMC9674857; doi:10.1038/s41598-022-24405-0)

Stacked kinship CNN vs. GBLUP for genomic predictions of additive and complex continuous phenotypes

Nelson Nazzicari[1,+] and Filippo Biscarini[2,+*]

[1]CREA: Council for Agricultural Research and Analysis of Agricultural Economics, Research Centre for Animal Production and Aquaculture, Viale Piacenza, 29 - 26900 Lodi
[2]CNR: National Research Council, Institute of Agricultural Biology and Biotechnology, Via Bassini 15, 20133 Milan, Italy
*filippo.biscarini@cnr.it
[+]these authors contributed equally to this work

Supplementary Figure S3: example of a training trajectory of the DNN (deep neural network) model. On the x-axis the training epochs. On the y-axis the metric of choice. Top panel: loss function (Mean Squared Error) used by the optimizer to tune the network. Bottom panel: Pearson correlation. Metrics are computed either on the training set (blue line) or the validation set (orange line).

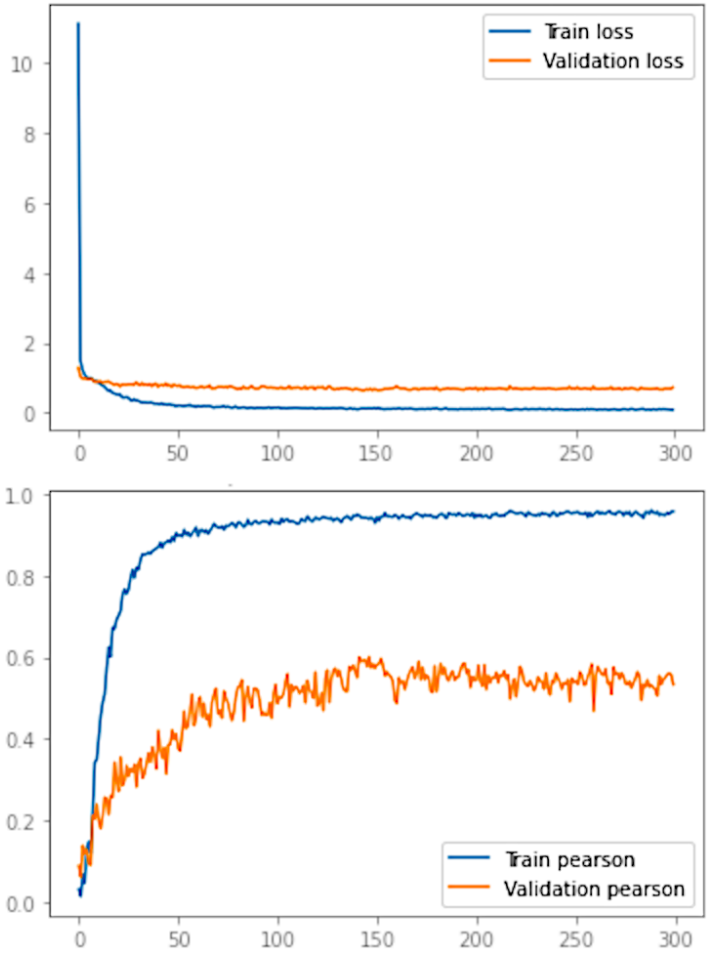

Supplement: Supplementary file 3 — Supplementary Figure 3. [file 41598_2022_24405_MOESM3_ESM.pdf]
