# Supplementary material for: Stacked kinship CNN vs. GBLUP for genomic predictions of additive and complex continuous phenotypes
Source: Sci Rep. 2022 Nov 18;12:19889. doi: 10.1038/s41598-022-24405-0 (PMC9674857; doi:10.1038/s41598-022-24405-0)
Supplement: Supplementary file 4 — Supplementary Table 1. [file 41598_2022_24405_MOESM4_ESM.pdf]

Stacked kinship CNN vs. GBLUP for genomic predictions of additive and complex continuous phenotypes

Nelson Nazzicari[1,+] and Filippo Biscarini[2,+*]

[1]CREA: Council for Agricultural Research and Analysis of Agricultural Economics, Research Centre for Animal Production and Aquaculture, Viale Piacenza, 29 - 26900 Lodi
[2]CNR: National Research Council, Institute of Agricultural Biology and Biotechnology, Via Bassini 15, 20133 Milan, Italy
*filippo.biscarini@cnr.it
[+]these authors contributed equally to this work

Supplementary Table S1: detailed summary of all the steps involved in building the deep learning model. For each hyperparameter that was considered, we report the values that we tried and the results obtained in terms of root mean squared error (RMSE) and Pearson correlation. The selected hyperparameter value is flagged with an ‘x’.

	Hyperparameter	RMSE	Pearson's correlation	Selected value
	**2dCNN kernel size**			
	3	0.645	0.510	**x**
	5	0.665	0.480	
	7	0.694	0.411	
	**padding**			
	valid	0.659	0.467	
	same	0.633	0.514	**x**
	**layer type (base architecture: 3x3 kernel, same padding)**			
3 layers	[8 16][32]	0.719	0.410	
	[16 32][64]	0.641	0.528	
	[16 64][32]	0.632	0.539	
	[32 64][16]	0.624	0.564	
	[32 64][128]	0.641	0.516	
	[64 128][256]	0.681	0.479	
4 layers	[8 16][8 4]	0.696	0.428	
	[8 16][16 8]	0.668	0.469	
	[8 16][64 32]	0.632	0.548	
	[16 32][16 8]	0.678	0.504	
	[16 32][32 16]	0.638	0.551	

	[16 32 64][8]	0.645	0.493	
	[16 32 64][16]	0.681	0.483	
	[16 32 64][32]	0.649	0.511	
	[16 32 64][64]	0.672	0.455	
	[32 64][16 8]	0.64	0.568	
	[32 64][32 16]	0.636	0.581	
	[32 64][64 32]	0.634	0.564	
	[32 64][128 64]	0.637	0.568	
	[32 32 64][16]	0.651	0.497	
	[32 64 64][16]	0.661	0.478	
	[64 128][32 16]	0.627	0.578	
	[64 128][64 32]	0.633	0.574	
5 layers	[16 32 64][16 8]	0.661	0.518	
	[32 64][32 16 8]	0.685	0.553	
	[32 64 128][64 32]	0.678	0.460	
	[32 32 64][32 16]	0.664	0.481	
	[32 32 64][64 32]	0.648	0.495	
	[32 64 64][64 32]	0.676	0.441	
	[32 64][32 16 8]	0.685	0.553	
	[32 64][64 32 16]	0.642	0.583	**x**
6 layers	[16 32 64][32 16 8]	0.648	0.514	
	[16 32 64][64 32 16]	0.666	0.514	
	[32 32 64][32 32 16]	0.642	0.529	
	[32 32 64][32 32 16]	0.666	0.503	
	**L1-L2 regularization (base architecture: 3x3 kernel, same padding, [32 64][64 32 16])**			
	no	0.642	0.583	**x**
	L1 = 0.0001 L2=0.0001	0.659	0.543	
	L1 = 0.0001 L2=no	0.701	0.465	
	L1 = 0.001 L2=0.001	0.771	0.279	
	L1 = 0.001 L2=no	0.796	0.241	
	L1 = 0.01 L2=no	0.764	0.291	
	L1 = no L2=0.0001	0.721	0.489	
	L1 = no L2=0.001	0.733	0.483	

	L1 = no L2=0.01	0.765	0.302	
	L1 = no L2=0.1	0.739	0.298	
	**Drop rate (base architecture: 3x3 kernel, same padding, [32 64][64 32 16])**			
	no	0.737	0.432	
	0.1	0.655	0.582	
	0.25	0.642	0.583	**x**
	0.4	0.746	0.549	
	0.5	0.799	0.221	
	**Data augmentation (base architecture: 3x3 kernel, same padding, [32 64][64 32 16], drop rate = 0.25)**			
	no	0.642	0.583	
	reps=1 sigma=0.01	0.658	0.597	
	reps=1 sigma=0.03	0.677	0.571	
	reps=1 sigma=0.05	0.660	0.577	
	reps=1 sigma=0.07	0.691	0.560	
	reps=1 sigma=0.09	0.690	0.554	
	reps=1 sigma=0.1	0.647	0.619	**x**
	reps=1 sigma=0.125	0.700	0.541	
	reps=1 sigma=0.15	0.687	0.512	
	reps=1 sigma=0.2	0.660	0.569	
	reps=1 sigma=0.3	0.641	0.614	
	reps=2 sigma=0.1	0.706	0.564	
	reps=2 sigma=0.3	0.654	0.537	
	reps=4 sigma=0.1	0.648	0.523	
	reps=4 sigma=0.3	0.651	0.574	
