# Supplementary material for: Stacked kinship CNN vs. GBLUP for genomic predictions of additive and complex continuous phenotypes
Source: Sci Rep. 2022 Nov 18;12:19889. doi: 10.1038/s41598-022-24405-0 (PMC9674857; doi:10.1038/s41598-022-24405-0)
Supplement: Supplementary file 5 — Supplementary Table 2. [file 41598_2022_24405_MOESM5_ESM.pdf]

Stacked kinship CNN vs. GBLUP for genomic predictions of additive and complex continuous phenotypes

Nelson Nazzicari[1,+] and Filippo Biscarini[2,+*]

[1]CREA: Council for Agricultural Research and Analysis of Agricultural Economics, Research Centre for Animal Production and Aquaculture, Viale Piacenza, 29 - 26900 Lodi
[2]CNR: National Research Council, Institute of Agricultural Biology and Biotechnology, Via Bassini 15, 20133 Milan, Italy
*filippo.biscarini@cnr.it
[+]these authors contributed equally to this work

Supplementary Table S2: detail of model performance for each model and phenotype. Predictive ability was measured as Pearson correlation, root mean squared error (RMSE) and normalized discounted cumulative gain (NDCG) on the top 20% of records. DNN: deep neural network model; GBLUP(A): GBLUP using only the additive kinship matrix; GBLUP-optimal: GBLUP using additive, dominant and epistasis kinship matrix as needed, depending on the phenotype.

regressor_flag	type_trait	trait	pearson_avg	pearson_std	rmse_avg	rmse_std	ndcg_avg	ndcg_std
DNN	A --> D	1_0_0_0_0	0.50511	0.05615	0.72318	0.05436	0.45242	0.13824
DNN	A --> D	0.75_0_0_0_0.25	0.51938	0.06564	0.70165	0.04466	0.44291	0.20244
DNN	A --> D	0.5_0_0_0_0.5	0.43695	0.07832	0.76065	0.04348	0.36591	0.16787
DNN	A --> D	0.25_0_0_0_0.75	0.30053	0.08773	0.81852	0.05508	0.26686	0.17729
DNN	A --> D	0_0_0_0_1	0.27388	0.05958	0.84647	0.03958	0.13848	0.21754
DNN	epistasis	0.33_0.34_0_0_0.33	0.41019	0.07519	0.76801	0.05041	0.34762	0.19856
DNN	epistasis	0.33_0.113_0.113_0.113_0.33	0.42592	0.08369	0.74506	0.04808	0.30816	0.24387
DNN	epistasis	0.33_0_0.34_0_0.33	0.40489	0.07536	0.76490	0.04272	0.37766	0.17080
DNN	epistasis	0.33_0_0_0.34_0.33	0.43392	0.05763	0.76891	0.04367	0.38019	0.19237
GBLUP_optimal	A --> D	1_0_0_0_0	0.66917	0.00537	0.74359	0.00400	0.65451	0.02412
GBLUP_optimal	A --> D	0.75_0_0_0_0.25	0.70611	0.00358	0.70805	0.00357	0.63553	0.04149
GBLUP_optimal	A --> D	0.5_0_0_0_0.5	0.61511	0.00779	0.78838	0.00586	0.54296	0.02746
GBLUP_op	A --> D	0.25_0_0_0_0.75	0.51287	0.01248	0.85788	0.00774	0.35366	0.03468

timal								
GBLUP_optimal	A --> D	0_0_0_0_1	0.50383	0.01826	0.86407	0.01056	0.22847	0.03357
GBLUP_optimal	epistasis	0.33_0.34_0_0_0.33	0.59749	0.00903	0.80259	0.00620	0.48326	0.03147
GBLUP_optimal	epistasis	0.33_0.113_0.113_0.113_0.33	0.60458	0.03249	0.79676	0.02486	0.48067	0.05220
GBLUP_optimal	epistasis	0.33_0_0.34_0_0.33	0.54567	0.00780	0.83892	0.00542	0.56090	0.03997
GBLUP_optimal	epistasis	0.33_0_0_0.34_0.33	0.62853	0.00884	0.77798	0.00651	0.54171	0.06139
GBLUP(A)	A --> D	1_0_0_0_0	0.67605	0.00595	0.73784	0.00528	0.65794	0.01953
GBLUP(A)	A --> D	0.75_0_0_0_0.25	0.70659	0.00730	0.70801	0.00725	0.63252	0.02812
GBLUP(A)	A --> D	0.5_0_0_0_0.5	0.58712	0.00444	0.81015	0.00242	0.58192	0.02319
GBLUP(A)	A --> D	0.25_0_0_0_0.75	0.37419	0.01330	0.92800	0.00671	0.46830	0.03046
GBLUP(A)	A --> D	0_0_0_0_1	0.28021	0.00915	0.96054	0.00327	0.46122	0.04276
GBLUP(A)	epistasis	0.33_0.34_0_0_0.33	0.56507	0.01265	0.82545	0.00955	0.51822	0.01487
GBLUP(A)	epistasis	0.33_0.113_0.113_0.113_0.33	0.58264	0.04718	0.81141	0.03405	0.49995	0.03994
GBLUP(A)	epistasis	0.33_0_0.34_0_0.33	0.51611	0.00775	0.85743	0.00424	0.58032	0.03980
GBLUP(A)	epistasis	0.33_0_0_0.34_0.33	0.60110	0.01098	0.79984	0.00822	0.58176	0.05213
