# Supplementary material for: Stacked kinship CNN vs. GBLUP for genomic predictions of additive and complex continuous phenotypes
Source: Sci Rep. 2022 Nov 18;12:19889. doi: 10.1038/s41598-022-24405-0 (PMC9674857; doi:10.1038/s41598-022-24405-0)
Supplement: Supplementary file 6 — Supplementary Table 3. [file 41598_2022_24405_MOESM6_ESM.pdf]

Stacked kinship CNN vs. GBLUP for genomic predictions of additive and complex continuous phenotypes

Nelson Nazzicari[1,+] and Filippo Biscarini[2,+*]

[1]CREA: Council for Agricultural Research and Analysis of Agricultural Economics, Research Centre for Animal Production and Aquaculture, Viale Piacenza, 29 - 26900 Lodi
[2]CNR: National Research Council, Institute of Agricultural Biology and Biotechnology, Via Bassini 15, 20133 Milan, Italy
*filippo.biscarini@cnr.it
[+]these authors contributed equally to this work

Supplementary Table S3: significance of differences between results from the GBLUP-A (benchmark), GBLUP-optim and DNN models for the prediction of the nine simulated phenotypes (from fully additive to fully phenotype, to different mixtures of epistatic effects). Results are shown in terms of Pearson correlation, root mean squared error (RMSE) and normalised discounted cumulative gain (NDCG). Estimates and significance of the differences come from analysis of variance. Traits (phenotypes) are encoded as: A_AA_AD_DD_D, where A stands for additive effects; AA, AD and DD stand for additive x additive, additive x dominance and dominance x dominance epistatic effects; D stands for dominant effect (1 indicates 100%, fractions refer to the relative proportions of the different types of genetic effects).

	trait	term	estimate	std.error	statistic	p.value
Pearson correlation	0_0_0_0_1	regressor_flagGBLUP-optim	0.2202828	0.0128348	17.1629748	3.20E-26
	0_0_0_0_1	regressor_flagDNN	-0.0054700	0.0117165	-0.4668601	0.6421145925
	0.25_0_0_0_0.75	regressor_flagGBLUP-optim	0.1271081	0.0191611	6.6336640	6.82E-09
	0.25_0_0_0_0.75	regressor_flagDNN	-0.0983258	0.0174916	-5.6213210	3.99E-07
	0.33_0_0_0.34_0.33	regressor_flagGBLUP-optim	0.0214497	0.0147076	1.4584139	0.1485462387
	0.33_0_0_0.34_0.33	regressor_flagDNN	-0.1438853	0.0124990	-11.5117241	8.34E-19
	0.33_0_0.34_0_0.33	regressor_flagGBLUP-optim	0.0332104	0.0168554	1.9703153	0.05259533941
	0.33_0_0.34_0_0.33	regressor_flagDNN	-0.1132503	0.0148650	-7.6185692	7.32E-11
	0.33_0.113_0.113_0.113_0.33	regressor_flagGBLUP-optim	0.0219171	0.0152513	1.4370680	0.1542854263
	0.33_0.113_0.113_0.113_0.33	regressor_flagDNN	-0.1395247	0.0152513	-9.1483909	2.23E-14
	0.33_0.34_0_0_0.33	regressor_flagGBLUP-optim	0.0222847	0.0174694	1.2756409	0.206487565
	0.33_0.34_0_0_0.33	regressor_flagDNN	-0.1352188	0.0159473	-8.4790882	3.30E-12
	0.5_0_0_0_0.5	regressor_flagGBLUP-optim	0.0308793	0.0175149	1.7630305	0.08196878271
	0.5_0_0_0_0.5	regressor_flagDNN	-0.1535058	0.0153008	-10.0325171	1.66E-15

	0.75_0_0_0_0.25	regressor_flagGBLUP-optim	-0.0001179	0.0159998	-0.0073709	0.9941396343
	0.75_0_0_0_0.25	regressor_flagDNN	-0.1563737	0.0142580	-10.9674507	6.20E-17
	1_0_0_0_0	regressor_flagGBLUP-optim	-0.0036860	0.0120816	-0.3050901	0.7612028528
	1_0_0_0_0	regressor_flagDNN	-0.1673625	0.0108266	-15.4585249	2.91E-24
	**trait**	**term**	**estimate**	**std.error**	**statistic**	**p.value**
RMSE	0_0_0_0_1	regressor_flagGBLUP-optim	-0.09445147	0.00843239	-11.20103438	5.31E-17
	0_0_0_0_1	regressor_flagDNN	-0.11458405	0.00769768	-14.88552616	6.27E-23
	0.25_0_0_0_0.75	regressor_flagGBLUP-optim	-0.06649955	0.01185631	-5.60878741	4.19E-07
	0.25_0_0_0_0.75	regressor_flagDNN	-0.09839732	0.01082328	-9.09126251	2.63E-13
	0.33_0_0_0.34_0.33	regressor_flagGBLUP-optim	-0.01653103	0.01109536	-1.48990537	0.1400856207
	0.33_0_0_0.34_0.33	regressor_flagDNN	-0.04761918	0.00942924	-5.05016183	2.62E-06
	0.33_0_0.34_0_0.33	regressor_flagGBLUP-optim	-0.02038436	0.00925645	-2.20217778	0.03081172937
	0.33_0_0.34_0_0.33	regressor_flagDNN	-0.09457130	0.00816343	-11.58475681	3.33E-18
	0.33_0.113_0.113_0.113_0.33	regressor_flagGBLUP-optim	-0.01425261	0.00956893	-1.48946777	0.1399805991
	0.33_0.113_0.113_0.113_0.33	regressor_flagDNN	-0.07834440	0.00956893	-8.18737667	2.06E-12
	0.33_0.34_0_0_0.33	regressor_flagGBLUP-optim	-0.01449867	0.01183097	-1.22548446	0.2246832156
	0.33_0.34_0_0_0.33	regressor_flagDNN	-0.07136193	0.01080015	-6.60749604	7.59E-09
	0.5_0_0_0_0.5	regressor_flagGBLUP-optim	-0.02419372	0.00977800	-2.47430052	0.01561018427
	0.5_0_0_0_0.5	regressor_flagDNN	-0.04702235	0.00854196	-5.50486830	4.94E-07
	0.75_0_0_0_0.25	regressor_flagGBLUP-optim	-0.00044305	0.01181206	-0.03750793	0.9701852817
	0.75_0_0_0_0.25	regressor_flagDNN	-0.03483299	0.01052611	-3.30919764	0.001471820071
	1_0_0_0_0	regressor_flagGBLUP-optim	0.00340998	0.01168356	0.29186162	0.7712556526
	1_0_0_0_0	regressor_flagDNN	-0.01710377	0.01046987	-1.63361828	0.1068291916
	**trait**	**term**	**estimate**	**std.error**	**statistic**	**p.value**

	0_0_0_0_1	regressor_flagGBLUP-optim	0.2327544672	0.04617589712	5.040605201	3.76E-06
	0_0_0_0_1	regressor_flagDNN	-0.08998911927	0.04215263411	-2.13483976	0.03643516421
	0.25_0_0_0_0.75	regressor_flagGBLUP-optim	0.1146312778	0.037694231	3.041082808	0.003362636025
	0.25_0_0_0_0.75	regressor_flagDNN	-0.08679965968	0.03440996767	-2.522515002	0.01403337529
	0.33_0_0_0.34_0.33	regressor_flagGBLUP-optim	0.01941522756	0.04048564858	0.4795582691	0.6328181177
	0.33_0_0_0.34_0.33	regressor_flagDNN	-0.1832427043	0.03440618535	-5.325865172	8.61E-07
	0.33_0_0.34_0_0.33	regressor_flagGBLUP-optim	0.01928710047	0.05444041712	0.3542790722	0.7241510759
	0.33_0_0.34_0_0.33	regressor_flagDNN	-0.1725011709	0.04801193499	-3.592881038	0.000590298373
NDCG	0.33_0.113_0.113_0.113_0.33	regressor_flagGBLUP-optim	0.04004867638	0.0310880757	1.288232722	0.2010806173
	0.33_0.113_0.113_0.113_0.33	regressor_flagDNN	-0.1615218481	0.0310880757	-5.195620649	1.33E-06
	0.33_0.34_0_0_0.33	regressor_flagGBLUP-optim	0.03495569536	0.04172408479	0.8377821956	0.4051317555
	0.33_0.34_0_0_0.33	regressor_flagDNN	-0.1356402954	0.03808870405	-3.56116856	0.0006850561173
	0.5_0_0_0_0.5	regressor_flagGBLUP-optim	0.03896106576	0.03772203868	1.032846238	0.3049949391
	0.5_0_0_0_0.5	regressor_flagDNN	-0.1770547166	0.03295356137	-5.372855291	8.40E-07
	0.75_0_0_0_0.25	regressor_flagGBLUP-optim	-0.003011879552	0.04440824918	-0.06782252415	0.9461175511
	0.75_0_0_0_0.25	regressor_flagDNN	-0.1926127853	0.03957364693	-4.867198255	6.61E-06
	1_0_0_0_0	regressor_flagGBLUP-optim	0.003432700408	0.02999505159	0.1144422238	0.9092148702
	1_0_0_0_0	regressor_flagDNN	-0.2020859182	0.02687915306	-7.518314204	1.41E-10
